# Hydrogen inhalation ameliorates ventilator-induced lung injury

**DOI:** 10.1186/cc9389

**Published:** 2010-12-25

**Authors:** Chien-Sheng Huang, Tomohiro Kawamura, Sungsoo Lee, Naobumi Tochigi, Norihisa Shigemura, Bettina M Buchholz, John D Kloke, Timothy R Billiar, Yoshiya Toyoda, Atsunori Nakao

**Affiliations:** 1 Department of Cardiothoracic Surgery, University of Pittsburgh Medical Center, 200 Lothrop Street, Pittsburgh, PA 15213, USA; 2 Division of Thoracic Surgery, Department of Surgery, Taipei-Veterans General Hospital and National Yang-Ming University School of Medicine, No.201 Sec.2, Shih-Pai Rd, Taipei, 112, Taiwan; 3 Thomas E Starzl Transplantation Institute, University of Pittsburgh Medical Center, 200 Lothrop Street, Pittsburgh, PA 15213, USA; 4 Department of Thoracic and Cardiovascular Surgery, Ajou University School of Medicine, San 5, Woncheon-dong, Youngtong-gu, Suwon, 443-749, South Korea; 5 Department of Pathology, University of Pittsburgh Medical Center, 200 Lothrop Street, Pittsburgh, PA 15213, USA; 6 Department of Medicine, Division of Gastroenterology and Hepatology, University of Pittsburgh Medical Center, 200 Lothrop Street, Pittsburgh, PA 15213, USA; 7 Center for Research on Health Care Data Center, University of Pittsburgh, 200 Lothrop Street, Pittsburgh, PA 15213, USA; 8 Department of Surgery, University of Pittsburgh Medical Center, 200 Lothrop Street, Pittsburgh, PA 15213, USA

## Abstract

**Introduction:**

Mechanical ventilation (MV) can provoke oxidative stress and an inflammatory response, and subsequently cause ventilator-induced lung injury (VILI), a major cause of mortality and morbidity of patients in the intensive care unit. Inhaled hydrogen can act as an antioxidant and may be useful as a novel therapeutic gas. We hypothesized that, owing to its antioxidant and anti-inflammatory properties, inhaled hydrogen therapy could ameliorate VILI.

**Methods:**

VILI was generated in male C57BL6 mice by performing a tracheostomy and placing the mice on a mechanical ventilator (tidal volume of 30 ml/kg without positive end-expiratory pressure, FiO_2 _0.21). The mice were randomly assigned to treatment groups and subjected to VILI with delivery of either 2% nitrogen or 2% hydrogen in air. Sham animals were given same gas treatments for two hours (*n *= 8 for each group). The effects of VILI induced by less invasive and longer exposure to MV (tidal volume of 10 ml/kg, 5 hours, FiO_2 _0.21) were also investigated (*n *= 6 for each group). Lung injury score, wet/dry ratio, arterial oxygen tension, oxidative injury, and expression of pro-inflammatory mediators and apoptotic genes were assessed at the endpoint of two hours using the high-tidal volume protocol. Gas exchange and apoptosis were assessed at the endpoint of five hours using the low-tidal volume protocol.

**Results:**

Ventilation (30 ml/kg) with 2% nitrogen in air for 2 hours resulted in deterioration of lung function, increased lung edema, and infiltration of inflammatory cells. In contrast, ventilation with 2% hydrogen in air significantly ameliorated these acute lung injuries. Hydrogen treatment significantly inhibited upregulation of the mRNAs for pro-inflammatory mediators and induced antiapoptotic genes. In the lungs treated with hydrogen, there was less malondialdehyde compared with lungs treated with nitrogen. Similarly, longer exposure to mechanical ventilation within lower tidal volume (10 mg/kg, five hours) caused lung injury including bronchial epithelial apoptosis. Hydrogen improved gas exchange and reduced VILI-induced apoptosis.

**Conclusions:**

Inhaled hydrogen gas effectively reduced VILI-associated inflammatory responses, at both a local and systemic level, via its antioxidant, anti-inflammatory and antiapoptotic effects.

## Introduction

Although ventilatory support is often required in the intensive care unit (ICU) for the treatment of critically ill patients with respiratory failure (including acute respiratory distress syndrome, pneumonia, septic shock, trauma, aspiration of vomit, and chemical inhalation), mechanical ventilation (MV) itself can induce lung injury and worsen preexisting lung injury depending on the setting and the length of ventilation [1,2]. This condition has been recognized as ventilator-induced lung injury (VILI). Despite recent progress in reducing the time on MV (for example, earlier weaning and extubation) and improving safety of MV (for example, lung protective ventilation with lower tidal volume), VILI remains a major concern in the ICU and can lead to remote organ dysfunction and multiple organ failure [3].

Multifactorial etiologies of VILI, from either direct or indirect injury to the lung, are postulated [4]. MV with high tidal volumes and pressure can lead to increased alveolar-capillary permeability accompanied by the release of pro-inflammatory mediators by the lung cells in response to mechanical stretch. These stimuli trigger detachment of endothelial cells from the basement membrane and synthesis of extracellular matrix components [5,6]. Injurious MV also promotes alveolar coagulopathy and fibrin deposition within the airways [7]. In addition, generation of reactive oxygen species (ROS) during VILI causes direct cellular injury and triggers ROS-sensitive, aberrant activation of cellular mechanisms leading to severe inflammation, resulting in rapid transcription of pro-inflammatory cytokines and chemokines [8,9].

Adjunctive therapy with inhaled therapeutic medical gas is promising and might be reasonable for lung disease as it would be an easily delivered and straightforward therapeutic option [10]. Hydrogen, recently discovered to be a novel therapeutic medical gas in a variety of biomedical fields, has potent antioxidant and anti-inflammatory efficacies by eliminating toxic ROS [11-14]. However, to our knowledge, hydrogen therapy has not been tested in the VILI setting. Though highly flammable, hydrogen is safe in concentrations of less than 4.6% when mixed with air and at concentrations of less than 4.1% when mixed with oxygen [15]. In this study, we investigated the hypothesis that, owing to its antioxidant and anti-inflammatory properties, inhaled hydrogen therapy could ameliorate VILI.

## Materials and methods

### Animals

Male wild-type C57BL6 mice (10 to 12 weeks old, 25 to 30 g) were purchased from The Jackson Laboratory (Bar Harbor, ME, USA). All animals were maintained in laminar flow cages in a specific pathogen-free facility at the University of Pittsburgh. The experimental protocol was approved by the Institutional Animal Care and Use Committee of the University of Pittsburgh, and all experiments were performed in adherence to the National Institutes of Health guidelines for the use of laboratory animals.

### Lung injury model

Mice were anesthetized by intraperitoneal injections of 85 mg/kg ketamine and 15 mg/kg xylazine. Then, under sterile conditions, a tracheostomy was performed with a 20-gauge angiocatheter which was sutured in place. Mice were placed in a supine position on a warming device and then connected to a ventilator (Harvard Apparatus, Holliston, MA, USA) on volume-control mode at a constant inspiratory flow. MV was initiated with a tidal volume of 30 mL/kg or 10 mL/kg (without an end-expiratory pressure) at a respiratory rate of 120 breaths per minute [16,17]. Mean arterial blood pressure was continuously monitored via catheterization of a femoral artery by means of a blood pressure monitor (Cardiomax III; Columbus Instruments, Columbus, OH, USA). Mice received intravenous injection of 0.05 mL/hour saline as well as intraperitoneal ketamine and xylazine to maintain the blood pressure at 75 to 80 mm Hg. At the end of the experiment, the animal was euthanized with 150 mg/kg ketamine intraperitoneally.

### Experimental design

Mice were randomly assigned to one of four experimental groups: MV (tidal volume 30 mL/kg, fraction of inspired oxygen [FiO_2_] 0.21, 2 hours) with 2% nitrogen in air (Praxair, Inc., Danbury, CT, USA), MV with 2% hydrogen in air (Praxair, Inc.), sham controls exposed to 2% nitrogen, or sham controls exposed to 2% hydrogen (*n *= 8 for each group). The concentration of 2% hydrogen was determined on the basis of previous observations as an optimal and safe concentration [11,18]. Mice under ventilation received the therapeutic (2% hydrogen) or control (2% nitrogen) gases via the tracheal tube. Animals in the sham groups were given therapeutic gases for 2 hours by means of a gas chamber [19] and underwent anesthesia only prior to sacrifice and procurement of tissue. While under anesthesia, the control mice received hydrogen or nitrogen through a face mask by spontaneous respiration. In separate experiments, mice were subjected to MV with a lower tidal volume for a longer period (10 mL/kg, FiO_2 _0.21, 5 hours) with 2% nitrogen or hydrogen in air (*n *= 6 for each group). At sampling, after the right lung was isolated and tied off with a microclamp at the right bronchus, the left lung was used for bronchoalveolar lavage (BAL). The right lower lobe was used for wet-to-dry (W/D) ratio measurement, the right middle lobe was used for histologic examination, and the other portions of the right lung were immediately snap-frozen in liquid nitrogen for further experiments, including gene expression analyses.

### Bronchoalveolar lavage

The left lung was used for BAL via slow intratracheal injection of three sequential 0.5-mL aliquots of sterile normal NaCl. Cell pellets obtained by centrifuging BAL samples at 1,500 rpm for 5 minutes at 4°C were resuspended in 1 mL of phosphate-buffered saline. The cell viability was determined via trypan blue exclusion assay. In brief, 10 μL of cells was mixed with 10 μL of 0.4% trypan blue and loaded onto a hemocytometer. Protein concentration in the bronchoalveolar lavage fluid (BALF) was measured with bovine IgG as a standard as previously described [20].

### Histopathological, immunohistochemistry, and TUNEL staining

For histologic evaluation, the right middle lobes of the lungs were fixed in 10% formalin, embedded in paraffin, sectioned to 6 μm in thickness, and stained with hematoxylin and eosin. The slides were blindly reviewed by one of the authors (NT) without knowledge of experimental groups (*n *= 6 for each group). Acute lung injury was scored according to the following four items: alveolar congestion, hemorrhage, infiltration or aggregation of neutrophils in the airspace or the vessel wall, and thickness of the alveolar wall/hyaline membrane formation [21]. For analysis of macrophage infiltration, formalin-fixed, paraffin-embedded mouse lung sections (4 μm) were deparaffinized and underwent antigen unmasking with an appropriate buffer. After protein blocking (DakoCytomation, Carpinteria, CA, USA) for 15 minutes, rat anti-mouse F4/80 primary antibody (clone: CI:A3-1; AbD Serotec, Raleigh, NC, USA) was applied and incubated overnight at 4°C. After blocking endogenous peroxidase, biotinylated goat anti-rat secondary antibodies (Jackson ImmunoResearch Laboratories, Inc., West Grove, PA, USA) were applied followed by ABC Elite reagent (Vector Laboratories, Burlingame, CA, USA). Staining was developed with AEC chromogen (ScyTek Laboratories, Inc., Logan, UT, USA), and the tissue was counterstained with hematoxylin. The TUNEL (terminal deoxynucleotidyl transferase-mediated deoxyuridine triphosphate nick-end labeling) method was used for identification of bronchiolar cell apoptosis with the ApopTag Peroxidase Kit (Intergen Co., Purchase, NY, USA). TUNEL-positive bronchial epithelial cells in five random, high-power fields per section were counted with the samples' identities masked.

### Arterial blood analysis

At the end of the experiment, arterial blood was obtained from the abdominal aorta. Blood gas analyses and measurement of lactate concentration were performed by means of an iSTAT handheld device (Abaxis, Union City, CA, USA).

### Wet-to-dry weight ratio

The right lower lobe was weighed immediately after collection and placed into a 60°C oven to dry for 2 days. The dried tissue was weighed to determine the W/D weight ratio.

### Real-time RT-PCR and tumor necrosis factor-alpha enzyme-linked immunosorbent assay

Since one of the underlying mechanisms of VILI is the release of pro-inflammatory mediators by the lung cells and airway epithelial cell apoptosis in response to mechanical stretch, quantitative real-time reverse transcription-polymerase chain reaction (RT-PCR) for inflammatory mediators was conducted on RNA extracted from the lung tissues. The mRNAs for early growth response-1 (Egr-1), chemokine (CC motif) ligand 2 (CCL2), interleukin (IL)-1β, tumor necrosis factor-alpha (TNFα), B-cell lymphoma-2 (*Bcl-2*), *Bcl-xL *(B-cell lymphoma-extra large), *Bax *(B-cell lymphoma-2-associated X-protein), and β-actin were quantified in duplicate by means of SYBR Green two-step, real-time RT-PCR as previously described [13]. The levels of serum TNF-α were detected by specific enzyme-linked immunosorbent assay (Thermo Scientific, part of Thermo Fisher Scientific Inc., Waltham, MA, USA) in accordance with the manufacturer's protocol.

### Malondialdehyde measurement

Oxidative injury from VILI was determined by measuring the tissue concentration of malondialdehyde (MDA), a marker of lipid peroxidation, by means of the MDA-586 kit (OxisResearch; Percipio Biosciences, Inc., Foster City, CA, USA) in accordance with the manufacturer's instructions.

### Statistical analysis

Results are presented as mean ± standard deviation. The EZAnalyze add-in for Microsoft Excel (Microsoft Corporation, Redmond, WA, USA) was used to perform analysis of variance with an F test and Bonferroni *post hoc *group comparisons. After the application of a log transformation to the data, Bartlett's test of homogeneity was not significant and comparison boxplots indicated that the assumption of constant variance was not violated. Nonetheless, in cases of possible heterogeneity, an analysis was performed on the log-transformed data. Histopathological score was analyzed with a Kruskal-Wallis test with *post hoc *Steel-Dwass test for group comparisons. A *P *value of less than 0.05 was considered statistically significant.

## Results

### Lung injury after mechanical ventilation with high tidal volume

**Figure 1:**
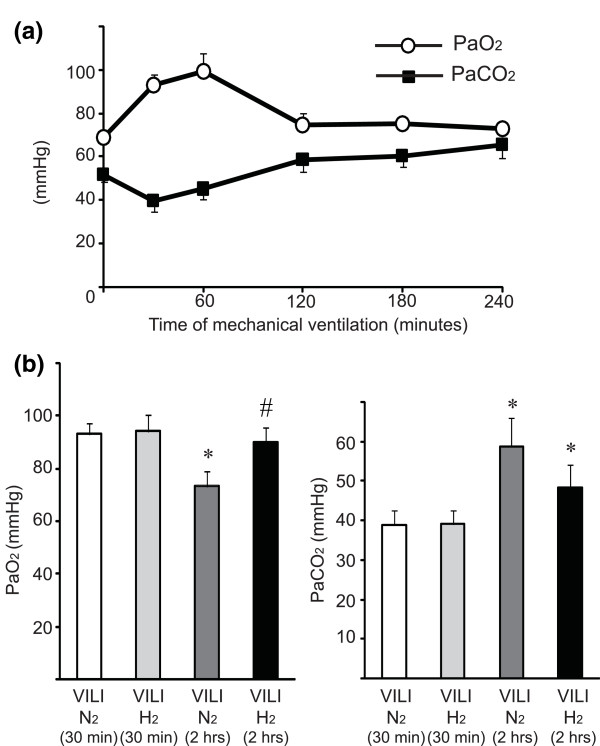
**Sequential blood gas analysis of mice exposed to mechanical ventilation with high tidal volume (30 mL/kg)**. **(a) **The impact of mechanical ventilation with 2% nitrogen in air on the lungs in a time-dependent manner was investigated. Animals showed deteriorated gas exchange before ventilation (0 hour) because of respiratory insufficiency caused by general anesthesia. Although mechanical ventilation improved gas exchange for the first hour, partial pressures of arterial oxygen (PaO_2_) were decreased with time and partial pressure of arterial carbon dioxide (PaCO_2_) increased with time. *N *= 3 to 6 for each time point. **(b) **Blood gas analysis for arterial blood after the end of 2 hours of mechanical ventilation with high tidal volume. There was improved pulmonary function in mice exposed to ventilator-induced lung injury (VILI) for 2 hours under 2% hydrogen compared with nitrogen controls. *N *= 8 for each group. *P < 0.05 versus VILI 30 minutes/N_2 _and VILI 30 minutes/H_2_; ^#^*P *< 0.05 versus VILI 2 hours/N_2_.

To evaluate the magnitude of lung injury by MV, sequential blood gas analysis of arterial blood from the mice exposed to 2% nitrogen via the ventilator was performed. Ventilator support improved gas exchange for the first 1 hour, while inhibition of gas exchange was seen in the animals at the beginning of MV because of muscle relaxation by general anesthesia. Subsequently, partial pressure of arterial oxygen (PaO_2_) gradually decreased with time in the ventilated mice and the partial pressure of arterial carbon dioxide (PaCO_2_) increased, suggesting that MV with high tidal volume provoked lung damage, related to alveolar overdistension or volutrauma, in a time-dependent manner (Figure 1a). To determine whether hydrogen inhalation affected hemodynamics, we monitored blood pressure and heart rate under MV. One hour after the start of MV, there was a significant decrease in mean arterial pressure in the mice ventilated with 30 mL/kg of tidal volume. There was no significant difference in hemodynamics between the VILI/N_2 _and VILI/H_2 _treatment groups over the 2-hour period. Inspiratory pressure was continuously monitored. Peak inspiratory pressure of the mice ventilated with 30 mL of tidal volume was 26.2 ± 0.7 cm H_2_O, which remained at constant levels throughout the experiment regardless of exposure to nitrogen or hydrogen.

### Gas exchange during ventilator-induced lung injury

Although the effects of MV with either 2% nitrogen or 2% hydrogen in air using a tidal volume of 30 mL/kg for 30 minutes on lung function were negligible, VILI was induced by 2 hours of ventilation with 2% N_2 _in air with a tidal volume of 30 mL/kg, as indicated by a significant decrease in PaO_2 _and an increase in PaCO_2_. Ventilation with 2% hydrogen in air exerted protective effects on the lungs and improved oxygenation of the arterial blood (Figure 1b). There was no statistical difference in PaCO_2 _levels among the groups. The blood pH did not differ between the 30-minute and 2-hour time points nor did it differ between the treatment groups (VILI/N_2 _for 30 minutes, blood pH 7.25 ± 0.05; VILI/H_2 _for 30 minutes, pH 7.28 ± 0.06; VILI/N_2 _for 2 hours, pH 7.24 ± 0.04; and VILI/H_2 _for 2 hours, 7.25 ± 0.05).

### Ventilator-induced lung injury-induced pulmonary edema

**Figure 2:**
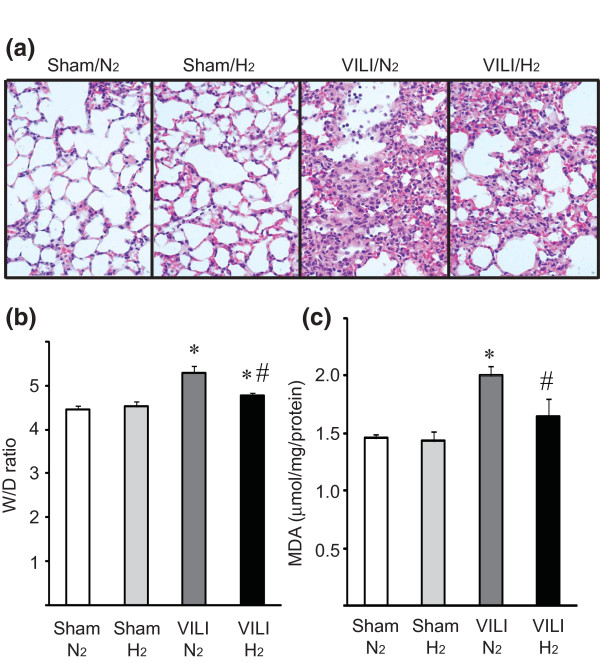
**Lung sections with mechanical ventilation with high tidal volume (30 mL/kg), stained with hematoxylin and eosin**. **(a) **Alveolar septal thickening and inflammatory cell infiltration were observed in the lung with ventilator-induced lung injury (VILI) (2% N_2_). Hydrogen administration markedly reduced these histopathological changes. Magnification = 400 ×. Representative images are shown. *N *= 6 animals for each experimental group. **(b) **Wet-to-dry (W/D) ratio of the lungs with mechanical ventilation with high tidal volume (30 mL/kg). VILI for 2 hours was accompanied with an increase of W/D ratio; ventilation with 2% hydrogen still induced lung edema but to a lesser extent compared with mechanical ventilation with 2% nitrogen in air. *N *= 6 for each group. **(c) **Tissue malondialdehyde (MDA) levels. Mechanical ventilation with high tidal volume (30 mg/kg) with 2% nitrogen in air increased tissue MDA levels. The supplementation of hydrogen significantly lowered levels of tissue MDA, a marker of lipid peroxidation. *N *= 6 for each group. *P < 0.05 versus sham/N_2 _and sham/H_2_; ^#^*P *< 0.05 versus VILI/N_2_.

MV exacerbated pulmonary inflammation and injury, as indicated by thickening of the alveolar septum and infiltration of inflammatory cells, evident in histopathological examination. In the presence of hydrogen, both edema and inflammatory cell infiltration were reduced despite exposure to MV with a 30 mL/kg tidal volume (Figure 2a) and the lung injury score was significantly improved with hydrogen inhalation (Table 1). Two hours of MV with high tidal volume (2% N_2 _in air) significantly increased the lung W/D ratio compared with lungs of sham mice. Ventilation with 2% hydrogen in air ameliorated ventilator-induced edema, as indicated by a significant decrease in lung W/D ratio as compared with ventilation with 2% N_2 _in air (Figure 2b).

**Table 1:** Lung injury scores after 2 hours of ventilator-induced lung injury (30 mL/kg)

		Lung injury score				
Group	Treatment	Alveolar congestion	Hemorrhage	Infiltration of neutrophils	Alveolar wall thickness	Total score
Sham	N_2_	0.25 ± 0.50	0	0.50 ± 0.58	0	0.75 ± 0.50
Sham	H_2_	0.25 ± 0.50	0	0.25 ± 0.50	0	0.50 ± 0.58
VILI	N_2_	1.75 ± 0.50	0	1.75 ± 0.96	1.50 ± 0.58	5.0 ± 1.63*
VILI	H_2_	0.75 ± 0.50	0	1.50 ± 0.57	0.75 ± 0.50	3.0 ± 0.82

Data are presented as mean ± standard deviation (* *P *< 0.05 versus ventilator-induced lung injury [VILI]/N_2_). Acute lung injury was scored in each sample (*n *= 6 for each group) according to the following four items: alveolar congestion, hemorrhage, infiltration or aggregation of neutrophils in airspace or the vessel wall, and thickness of the alveolar wall/hyaline membrane formation. Each item was graded according to a 5-point scale: 0, minimal (little) damage; 1, mild damage; 2, moderate damage; 3, severe damage; and 4, maximal damage.

### Lung lipid peroxidation

Although there were increased MDA-protein adducts in the lung ventilated with a high tidal volume of 2% nitrogen in air, ventilation with 2% hydrogen reduced tissue MDA levels after 2 hours of MV with high tidal volume (Figure 2c).

### Alveolar-capillary leak due to ventilator-induced lung injury

**Figure 3:**
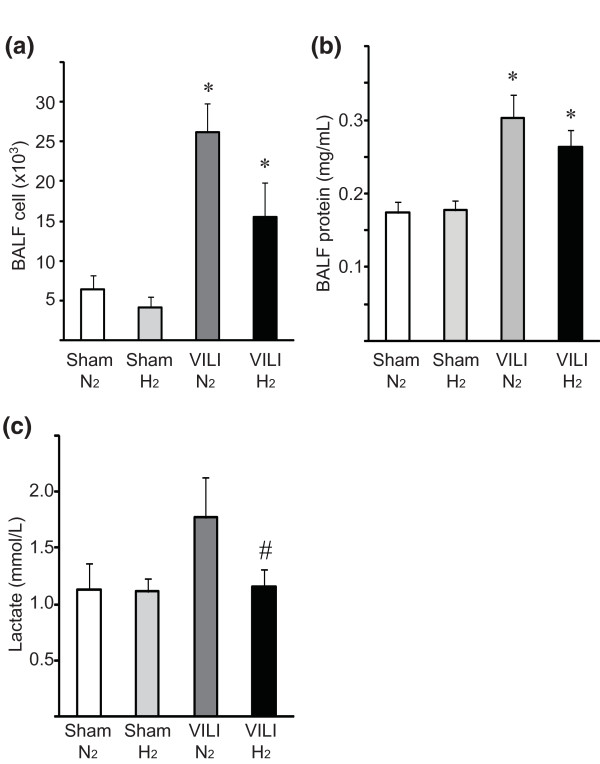
**Number of infiltrating cells recovered in the bronchoalveolar lavage fluid (BALF) obtained from the lungs with mechanical ventilation with higher tidal volume (30 mL/kg)**. **(a) **Administration of hydrogen had no effect on inflammatory cell accumulation in the BALF. *N *= 6 for each group. ^#^*P *< 0.05 versus sham/N_2 _controls. **(b) **Protein concentration in BALF. Ventilator-induced lung injury (VILI) resulted in significant increases of protein contained in BALF. Hydrogen did not reduce leaked protein. *N *= 6 for each group. **(c) **Blood lactate concentration. VILI by mechanical ventilation for 2 hours was associated with hyperlactatemia. Mice subjected to ventilation with 2% hydrogen did not show significant increase of blood lactate levels compared with those of sham controls. *N *= 6 for each group. *P < 0.05 versus sham/N_2 _and sham/H_2_; ^#^*P *< 0.05 versus VILI/N_2_.

MV with 30 mL/kg tidal volume caused an acute exudative phase with alveolar-capillary leak in conjunction with leukocyte extravasation and resulted in an increase in the total cell number in the BALF. The effects of hydrogen on total cells or protein concentration in the BALF were marginal (Figure 3a, b). While blood lactate levels increased in ventilated mice receiving 2% nitrogen, they did not increase in mechanically ventilated mice receiving 2% hydrogen in air (Figure 3c).

### Expression of inflammatory mediators and apoptosis-related genes

**Figure 4:**
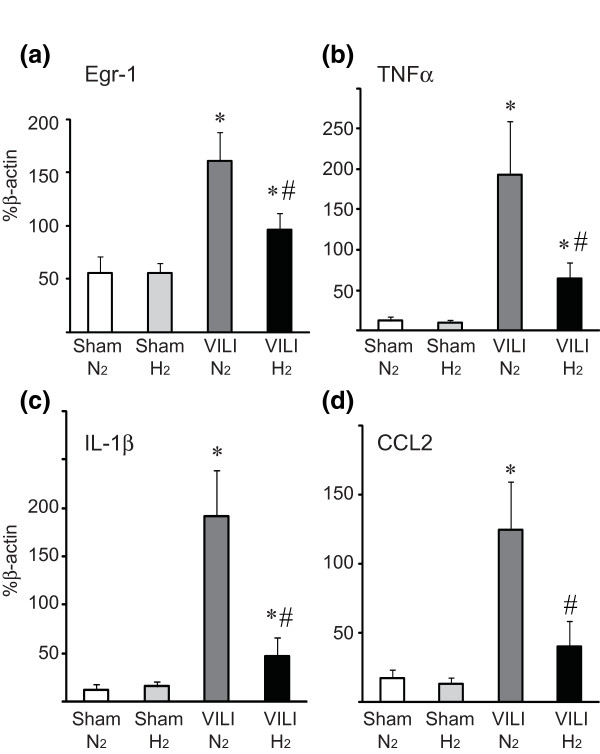
**Quantitative reverse transcription-polymerase chain reaction for inflammatory mediators and transcripts in lung tissues with mechanical ventilation with higher tidal volume (30 mL/kg)**. The levels of mRNAs for early growth response-1 (Egr-1) **(a)**, tumor necrosis factor-alpha (TNFα) **(b)**, interleukin-1-beta (IL-1β) **(c)**, and CCL-2 **(d) **significantly increased after mechanical ventilation with either with 2% nitrogen in air (VILI/N_2_) or 2% hydrogen in air (VILI/H_2_). However, mRNA expression was significantly less in the VILI/H_2 _group compared with the VILI/N_2 _group. All values are reported as percentage of β-actin with normalization to β-actin mRNA expression. *N *= 8 for each group. *P < 0.05 versus sham/N_2 _and sham/H_2_; ^#^*P *< 0.05 versus VILI/N_2_. CCL2, chemokine (CC motif) ligand 2; VILI, ventilator-induced lung injury.

**Figure 5:**
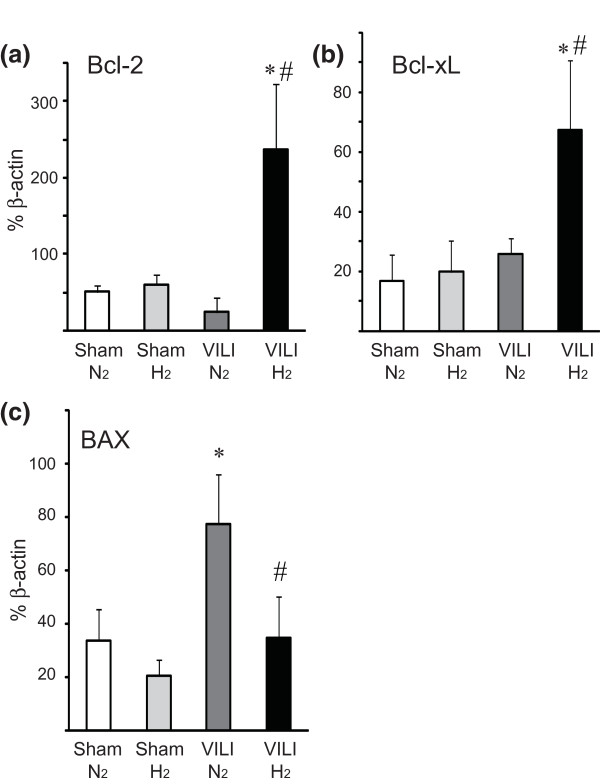
**Quantitative reverse transcription-polymerase chain reaction for apoptosis-related genes in lung tissues with mechanical ventilation of higher tidal volume (30 mL/kg)**. The levels of mRNAs for *Bcl-2 ***(a) **and *Bcl-xL ***(b) **significantly increased after mechanical ventilation in the presence of 2% hydrogen in air (VILI/H_2_). On the other hand, *Bax ***(c) **was significantly increased after mechanical ventilation with 2% nitrogen in air (VILI/N_2_) and was not increased after mechanical ventilation with 2% hydrogen in air. All values are reported as percentage of β-actin with normalization to β-actin mRNA expression. *N *= 6 for each group. *P < 0.05 versus sham/N_2 _and sham/H_2_; ^#^*P *< 0.05 versus VILI/N_2_. *Bcl-2*, B-cell lymphoma-2; *Bcl-xL*, B-cell lymphoma-extra large; VILI, ventilator-induced lung injury.

VILI after ventilation with 2% nitrogen resulted in upregulation of mRNAs for Egr-1, TNFα, IL-1β, and CCL2. Hydrogen administration significantly attenuated the upregulation of the mRNAs for these inflammatory mediators (Figure 4). Hydrogen inhalation increased the expression of antiapoptotic genes, such as *Bcl-2 *and *Bcl-xL*, and reduced VILI-induced expression of the pro-apoptotic Bax gene (Figure 5). There was no significant difference in mRNA expression for the housekeeping gene β-actin among the groups (data not shown).

### Ventilator-induced lung injury with lower tidal volume

**Figure 6:**
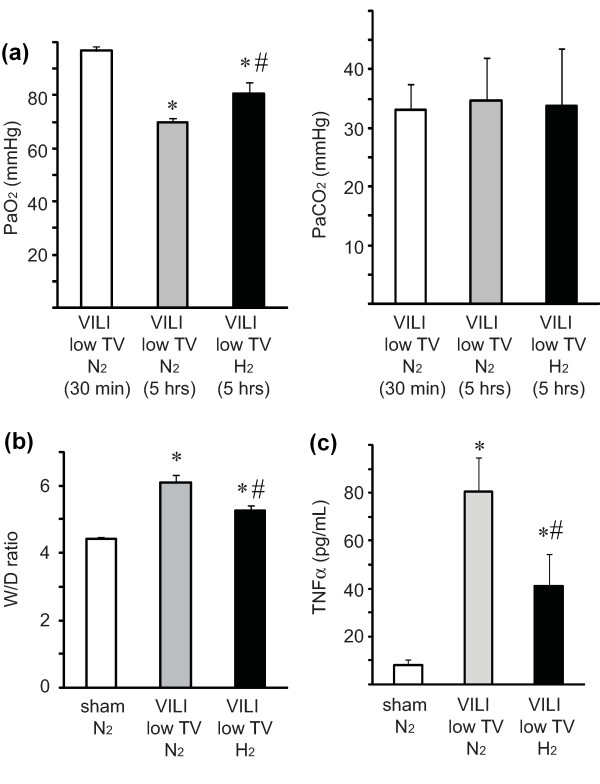
**The effects of hydrogen on ventilator-induced lung injury (VILI) induced by mechanical ventilation with low tidal volume (TV) (10 mL/kg)**. **(a) **Partial pressure of arterial oxygen (PaO_2_) levels of mice ventilated with 2% hydrogen for 5 hours were significantly higher than those of mice ventilated with 2% nitrogen. *N *= 6 for each group. **(b) **Lung edema caused by VILI was determined by measuring the wet-to-dry (W/D) ratio. Hydrogen inhalation therapy ameliorated VILI-induced lung edema caused by mechanical ventilation with low TV. *N *= 6 for each group. **(c) **Serum tumor necrosis factor-alpha (TNFα) concentrations were measured after VILI with TV of 10 mL/kg for 5 hours. Hydrogen treatment significantly reduced VILI-induced elevation of serum TNFα. *N *= 6. *P < 0.05 versus sham/N_2 _and sham/H_2_; ^#^*P *< 0.05 versus VILI/N_2_.

Finally, we analyzed whether hydrogen therapy could also attenuate VILI induced using a less invasive protocol with longer exposure to MV of lower tidal volume. Inducing VILI in the mice via a tidal volume of 10 mL/kg for 5 hours resulted in deterioration of gas exchange in mice receiving 2% nitrogen with an associated increase in pulmonary edema. These lung injuries were significantly attenuated by treatment with 2% hydrogen (Figure 6a, b). Hydrogen treatment significantly reduced serum TNFα concentrations as compared with the serum levels of TNFα in mice with VILI caused by ventilation with 2% nitrogen (Figure 6c).

**Figure 7:**
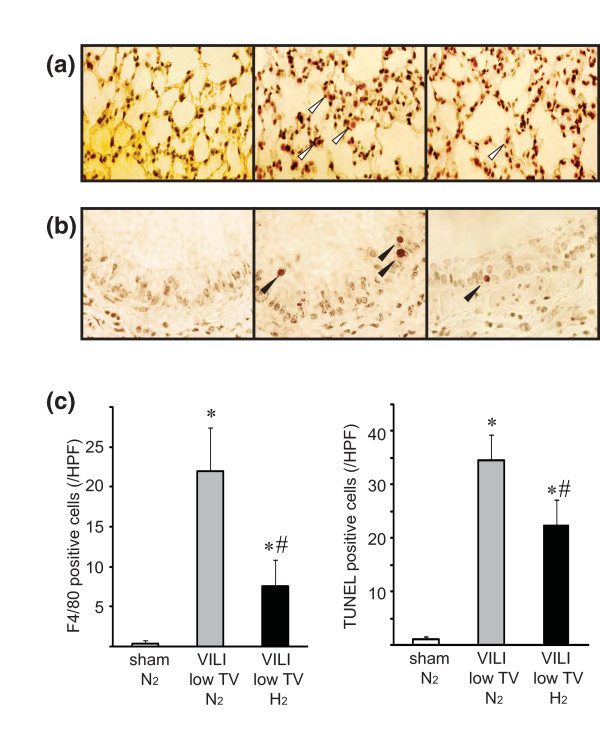
**The effects of hydrogen on ventilator-induced lung injury (VILI)-associated histopathological changes by mechanical ventilation with low tidal volume (10 mL/kg)**. **(a) **Representative images of lungs stained with F4/80 to visualize macrophages. Left panel: There were few F4/80-positive cells in the lungs of sham control mice. Middle panel: A marked increase in F4/80-postive cells was observed in the lung with low-tidal volume ventilation. Right panel: Ventilation with 2% hydrogen significantly reduced the number of macrophages. Magnification = 600 ×. Representative images from four animals for each experimental group are shown. White arrowheads indicate F4/80-positive cells. **(b) **Bronchial cell apoptosis was determined by TUNEL (terminal deoxynucleotidyl transferase-mediated deoxyuridine triphosphate nick-end labeling) assay. Left panel: Few apoptotic cells were seen in the bronchial epithelium of sham control mice. Middle panel: There was an increase in the number of TUNEL-positive cells in the bronchial epithelium in the lung with VILI in 2% nitrogen. Right panel: Hydrogen therapy decreased the number of TUNEL-positive cells. Magnification = 800 ×. Representative images from four animals for each experimental group are shown. Black arrowheads indicate TUNEL-positive cells. **(c) **The positive cells were counted in a blinded manner and expressed as the number per high-power field (HPF). Histograms indicate the number of F4/80-positive cells/HPF and TUNEL-positive epithelial cells/HPF. *N *= 4. *P < 0.05 versus sham/N_2 _and sham/H_2_; ^#^*P *< 0.05 versus VILI/N_2_.

In this VILI model with lower tidal volume, hydrogen treatment significantly decreased macrophage infiltration, as determined by F4/80 staining, as compared with lungs ventilated with 2% nitrogen (Figure 7a, c). MV with 2% nitrogen also increased apoptotic cell death of bronchial epithelial cells, as determined by TUNEL. Ventilation with 2% hydrogen significantly reduced TUNEL-positive epithelial cells as compared with ventilation with 2% nitrogen (Figure 7b, c).

## Discussion

In this study, we demonstrated that administration of hydrogen gas mitigated VILI and VILI-associated oxidative and inflammatory responses as well as VILI-induced apoptotic cell death of bronchial epithelial cells. To our knowledge, this is the first study to demonstrate that hydrogen gas significantly reduces VILI. Since VILI is a major concern with intensive care, approaches to minimize VILI will advance critical care medicine and could have substantial clinical impact.

Recently, the biological functions of therapeutic gases have received considerable attention and hydrogen was identified as a physiologically relevant gaseous signaling molecule like other endogenously generated gases, including nitric oxide (NO), carbon monoxide, and hydrogen sulfide [22-24]. Thus, hydrogen has been described as 'the fourth signaling gaseous molecule' [25].

Hydrogen has great potential as a safe and potent therapeutic medical gas as well as several potential advantages as a therapeutic option for VILI. Inhalation therapy is a straightforward approach to lung disease and can be administered by simply providing gas for the patient to inhale. Hydrogen may be relatively easily incorporated into our current interventional or surgical procedures without increasing their complexity. Inhaled hydrogen gas has been safely used for treatment of decompression syndrome in divers [26], suggesting that hydrogen can be safely administered to patients. Hydrogen is a stable molecule and does not react with other therapeutic medical gases at room temperature and thus may be administered as a combined gas with other therapeutic gases or inhaled anesthesia agents [18]. Hydrogen does not alter NO levels [11]. Endogenous NO signaling pathways are critical for modulating pulmonary vascular tone and leukocyte/endothelial interactions; therefore, it may be beneficial to spare endogenous NO [27]. Hydrogen treatment does not eliminate superoxide anion (O_2_^-^) or hydrogen peroxide (H_2_O_2_) [11]. O_2_^- ^and H_2_O_2 _have important functions in neutrophils and macrophages, allowing phagocytosis. Hydrogen therapy may spare the innate immune system, and this would be beneficial because lung infection accompanies VILI in many cases [28].

Importantly, experimental studies have demonstrated the protective effects of hydrogen for septic shock [29], brain injury [11], liver injury [12], ischemic heart disease [30], and paralytic ileus [13]. As all of these diseases frequently coincide with VILI in ICU patients, inhaled hydrogen therapy by simply delivering the gas through the ventilator could be a very promising adjunctive therapy in the ICU or operating room.

In our study, hydrogen inhalation ameliorated upregulation of the mRNAs for TNFα, IL-1β, Egr-1, and CCL2 after 2 hours of MV, and this may explain the anti-inflammatory mechanisms afforded by hydrogen in this VILI model. Egr-1 acts as a key pro-inflammatory regulator in VILI. Hoetzel and colleagues [31] demonstrated that Egr-1-deficient mice did not sustain lung injury after ventilation, relative to wild-type mice. The CC chemokine family is essential for the leukocyte recruitment during inflammation. Mounting evidence suggests that CCL2, a member of the CC chemokine family, is involved in numerous inflammation disorders of the lung, including VILI [32]. Pro-inflammatory cytokines, such as TNFα and IL-1β, are elevated and play pivotal roles during the pathogenesis of VILI [33].

In our model, the increase in W/D ratio because of MV was relatively mild, despite the larger changes observed in lung function (gas exchange). The histopathology changes were moderate. We are unsure of the reason for these discrepancies, although perhaps W/D ratio is a very sensitive method to detect lung edema and a small difference of W/D ratio may represent significant edema. The gravimetric measure of lung edema poses considerable technical challenges, including evaporative loss and regional heterogenesity. W/D can be complicated by the inclusion of blood in the wet lung weight from both residual intravascular blood and blood introduced into the lung interstitium via bleeding or injury [34]. In addition, extravasated protein can contribute to total lung weight. Although there are mismatches in magnitude of lung injury depending on which parameter is evaluated, each evaluation is scientifically sound and each indicates some degree of VILI that was ameliorated by hydrogen treatment.

VILI has been shown to induce apoptosis of airway epithelial cells [35]. We demonstrated, in the present study, that hydrogen could upregulate antiapoptotic genes, including *Bcl-2 *and *Bcl-xL*. Although our findings do not explain all of the mechanisms underlying the protective effects of hydrogen, we postulate that the *Bcl-2*/*Bcl-xL *pathway might be one of the key mechanisms. Additionally, cyclic stretch associated with high-tidal-volume MV generates ROS and redox imbalance in lung epithelial and endothelial cells [36]. The antioxidant properties of hydrogen to eliminate ROS may contribute to the mitigation of VILI in our model.

In our blood gas analyses, there were no significant differences in PaCO_2 _levels between animals exposed to 2% nitrogen or 2% hydrogen, although MV for 2 hours significantly increased PaCO_2 _levels compared with MV for 30 minutes. These findings suggest that PaCO_2 _levels may not be the best therapeutic indicator in our model, although PaO_2 _levels are very sensitive indicators of the magnitude of VILI. Our ventilation protocol was based on maintaining PaCO_2 _in a range of 25 to 35 mm Hg during the first 30 minutes of the MV period. We acknowledge the limitations of using PaCO_2 _as a marker of VILI in our model; as a marker, PaCO_2 _would not be applicable for clinical use and may be influenced by auto-positive end-expiratory pressure, overdistension, or atelectasis; however, the PaCO_2 _changes observed in our study were likely caused by VILI and not by a poorly controlled MV setting.

PaCO_2 _levels can influence inflammation and edema of the lungs. Hypocapnia increases microvascular permeability and impairs alveolar fluid reabsorption, which may influence the pathogenesis of pulmonary edema [37]. Furthermore, hypocapnia is directly injurious to lung parenchyma and worsens ischemia/reperfusion injury [38]. Therefore, permissive hypercapnia has been proposed as a protective strategy during MV and contributes to the attenuation of lung inflammatory response and pulmonary edema [39-42], and the potential exists for an independent protective/pathological role of alterations in carbon dioxide tension in the context of VILI. However, the VILI model in this study was designed to be normocapnic, and our results demonstrated that higher PaCO_2 _levels were not associated with less lung inflammation or edema, suggesting no influence of PaCO_2 _levels in our VILI model.

A more comprehensive understanding of the pharmacokinetics, biology, and toxicity of hydrogen will certainly help us harness the protective potential of hydrogen gas prior to clinical application. Virtually all patients under ventilation receive oxygen, and, in particular, patients with pulmonary disease usually require high levels of oxygen. Although hydrogen, when present at concentrations of less than 4%, poses no risk of explosion in air and oxygen, safety is still a concern, and the desired concentration of hydrogen must be legitimately monitored and maintained with commercially available tools. In this study, no adverse events related to hydrogen were observed. We acknowledge that this experimental model, like most animal models, does not recapitulate all aspects of clinical VILI. However, it is a scientifically sound study using a model appropriate to address the hypothesis. These findings will serve as a springboard to further translational research. Although extensive studies on toxicity and safety are needed, hydrogen treatment of ventilated patients may be clinically feasible and would be easy to incorporate without alteration of interventional and surgical procedures.

## Conclusions

This study demonstrated a novel anti-inflammatory, antioxidative, and antiapoptotic function of hydrogen that ameliorated MV-induced lung injury.

## Key messages

• Hydrogen inhalation therapy at a safe concentration mitigated ventilator-induced lung injury in mice.

• Hydrogen inhalation demonstrated potent antioxidant and anti-inflammatory effects in a mouse ventilator-induced lung injury model.

• Hydrogen reduced ventilation-induced epithelial apoptosis by induction of antiapoptotic genes.

• Adjunctive therapy with inhaled therapeutic medical gas is promising and might be reasonable for lung disease as it would be an easily delivered and straightforward therapeutic option.

• Hydrogen treatment of ventilated patients may potentially yield a novel, clinically feasible therapy that would be easy to incorporate without alteration of interventional and surgical procedures.

## Abbreviations

BAL: bronchoalveolar lavage; BALF: bronchoalveolar lavage fluid; *Bax*: B-cell lymphoma-2-associated X-protein; *Bcl-2*: B-cell lymphoma-2; *Bcl-xL*: B-cell lymphoma-extra large; CCL2: chemokine (CC motif) ligand 2; Egr-1: early growth response-1; FiO_2_: fraction of inspired oxygen; H_2_O_2_: hydrogen peroxide; ICU: intensive care unit; IL: interleukin; MDA: malondialdehyde; MV: mechanical ventilation; NO: nitric oxide; O_2_^-^: superoxide anion; PaCO_2_: partial pressure of arterial carbon dioxide; PaO_2_: partial pressure of arterial oxygen; ROS: reactive oxygen species; RT-PCR: reverse transcription-polymerase chain reaction; TNFα: tumor necrosis factor-alpha; TUNEL: terminal deoxynucleotidyl transferase-mediated deoxyuridine triphosphate nick-end labeling; VILI: ventilator-induced lung injury; W/D: wet-to-dry.

## Competing interests

The authors declare that they have no competing interests.

## Authors' contributions

C-SH initiated the experimental design, participated in animal study, and drafted the manuscript. TK and SL carried out the molecular genetic studies and participated in the sequence alignment. NS, TRB, and YT participated in study design and coordination and helped to draft the manuscript. NT contributed to histopathological analysis. BMB and JDK contributed to statistical analysis. AN provided the working hypothesis, designed the study, helped to conduct the research, and wrote the manuscript. All authors read and approved the final manuscript.
